# Behavioral resilience via dynamic circuit firing homeostasis

**DOI:** 10.1073/pnas.2421386122

**Published:** 2025-04-29

**Authors:** Adam Hoagland, Ryan Schultz, Zerong Cai, Zachary L. Newman, Ehud Y. Isacoff

**Affiliations:** ^a^Department of Molecular and Cell Biology, University of California Berkeley, Berkeley, CA 94720; ^b^Helen Wills Neuroscience Institute, University of California Berkeley, Berkeley, CA 94720; ^c^Department of Neuroscience, University of California Berkeley, Berkeley, CA 94720; ^d^Weill Neurohub, University of California Berkeley, Berkeley, CA 94720; ^e^Molecular Biophysics and Integrated BioImaging Division, Lawrence Berkeley National Laboratory, Berkeley, CA 94720

**Keywords:** homeostasis, synapse, glutamatergic, locomotion, motor neuron

## Abstract

Homeostatic regulation ensures stable neural circuit output under changing conditions. We find that in *Drosophila* larvae, either presynaptic weakening due to perturbation of transmitter release or postsynaptic weakening due to perturbation of glutamate receptors at synapses between motor neuron (MN) and muscle has little impact on locomotion, suggesting a nonsynaptic compensatory mechanism. In vivo imaging shows that five different forms of synaptic weakening increase the duration of activity bouts in type I MNs. Strikingly, this compensation is input selective: occurring only in the tonic type Ib MN, not the phasic type Is MN that innervates the same muscle. Moreover, an inhibitory class of central pre-MNs that innervates the tonic—but not phasic—input decreases in activity. The adjustment in activity occurs remarkably quickly: within minutes of synapse perturbation. We propose that MN firing is dynamically regulated by two coordinated mechanisms: a cell-autonomous adjustment of MN excitability and a circuit adjustment of inhibitory central drive. The input selectivity of this process suggests homeostatic adjustment to maintain tonic drive but hold constant the phasic drive that organizes locomotory wave patterns.

## Significance

As animals navigate the world, plasticity and perturbations change the strength of specific synaptic connections between neurons. To prevent these changes from throwing off circuit operations, homeostatic adjustment returns overall transmission into balance. In the *Drosophila* motor system, presynaptic homeostasis compensates for a postsynaptic defect, but only partially, and yet, we find locomotion to be normal. This suggests that a second form of homeostasis may also exist. Indeed, we find that when the synapse between motor neurons and muscle weakens, motor neurons compensate by increasing firing due to a combined effect of increased excitability and reduced inhibitory central drive. Firing homeostasis occurs in one of two motor neurons, providing flexibility, while the nonhomeostatic motor neuron ensures constancy.

Synaptic plasticity is a fundamental property of neurons that allows them to modify their outputs in response to external cues, shaping neuronal circuits and influencing behavior. Yet such flexibility faces remarkable regulatory challenges throughout the lifespan. Through development, synaptic connections undergo significant structural and functional changes, including extensive growth, pruning, and refinement. At the same time, neurons experience fluctuations in intrinsic excitability by rapidly increasing in size and altering channel abundances and distributions. Once maturation is reached, networks must retain plasticity and be amenable to experience-dependent synaptic strengthening mechanisms, such as short- or long-term potentiation, which are crucial for learning and memory (1–3). Yet left unchecked, these activity-dependent, or Hebbian, forms of synaptic strengthening have the potential to create positive feedback loops and runaway excitation, which can propagate through circuits and lead to network instability (4). Furthermore, synapses must sustain consistent and reliable signaling, even as their molecular machinery—such as ion channels, receptors, and signaling proteins—undergoes constant replacement (5–8). Therefore, for an animal’s behavior to be both adaptable and consistent, counteracting forces must coexist that generate flexibility and sustain stability.

The nervous system accomplishes this balance by employing various homeostatic plasticity mechanisms that stabilize circuit function and regulate cell excitability. Such adjustments return cells to firing setpoints (9), restore excitation–inhibition balance (10), regulate neuronal intrinsic excitability (5, 11), and ensure reliable circuit output by adjusting synapses both presynaptically and postsynaptically (12–14). The two most characterized forms of homeostatic synaptic plasticity include synaptic scaling and presynaptic homeostatic plasticity (PHP). Synaptic scaling works by uniformly scaling the strengths of all synapses, either up or down, in order bring the neuron’s overall activity back to within target range while preserving the relative strength of synapses (15–17). PHP, which has largely been characterized at the *Drosophila* neuromuscular junction (NMJ), compensates for weakening of the postsynaptic response via a presynaptic boost in transmitter release (18–22). Of long-standing interest is the time course over which different homeostatic responses can occur. Synaptic scaling occurs over hours to days (15–17), acute PHP over minutes (19), and intrinsic excitability adjustment within seconds (23). These adjustments can persist for days to years (24–27). Yet while various homeostatic mechanisms have been explored in different model organisms, few studies investigate a system that integrates multiple forms of these mechanisms simultaneously—particularly within a behaving animal with an intact nervous system (28). Such investigation would provide valuable insight into the coordinated function of multiple homeostatic adjustments, while also enabling the examination of the timing of these responses following perturbations to the animal’s nervous system.

In this paper, we present an analysis of the behaving *Drosophila* larva with an intact nervous system, centered around adjustments in the dynamics of firing of motor neurons and their inputs in response to acute and chronic perturbations to motor neuron to muscle transmission. The *Drosophila* larval neuromuscular junction features two classes of type I glutamatergic motor neurons (MNs): tonic type Ib MNs, which innervate individual muscles, and phasic type Is MNs, which innervate multiple muscles within a group (29). Both Ib and Is inputs provide approximately half of the excitatory drive to the muscle (30). Under physiological conditions, when transmission is weakened by mutation of the GluRIIA subunit of the GluRII postsynaptic ionotropic glutamate receptor or by partial pharmacological block of GluRII, a retrograde signal from the muscle triggers the compensatory PHP boost in glutamate release (18, 19, 31). Although PHP compensates completely for reduced postsynaptic response under pharmacological block of GluRIIA, it is incomplete in the GluRIIA mutant in both the Ib and Is (32, 33). This shortfall in synaptic compensation leaves a weakened overall neuromuscular transmission. We hypothesized that this perturbation would result in altered locomotion. However, locomotion in the GluRIIA mutant larvae is almost indistinguishable from that of wild-type animals.

To understand how locomotion could be preserved when synaptic transmission is weakened postsynaptically and only partially compensated presynaptically, we turned to more severe presynaptic perturbations. We examined locomotion in larvae with severely reduced glutamate release due to knockdown of three key components of the MN transmitter release machinery: i) the voltage-gated Ca2+ channel, Cac, which couples the action potential (AP) to release, ii) the synaptic vesicle priming protein Unc-13, and iii) RIM binding protein (Rbp). Each knockdown greatly reduced the average probability of AP-evoked release (Pr) across the population of synapses, leading us to expect a major disruption in locomotion. However, once again, locomotion was near normal in these knockdown animals. This suggested the presence of an additional homeostatic mechanism that preserves behavior. Further investigation revealed that weakened transmission led to increased duration of MN activity and synaptic transmission, particularly in type Ib MNs, suggesting an increase in MN excitability. Knockdowns of K_v_ channels in type I MNs supported this hypothesis. Additionally, we measured reduced activity in a population of inhibitory premotor neurons, consistent with our observed increase in MN activity. These findings demonstrate that homeostatic adjustments in type Ib MNs, involving both increased excitability and reduced inhibitory input, preserve locomotion when neuromuscular transmission is compromised.

## Results

### Screen to Identify Presynaptic Deficiency in Neurotransmission at Ib and Is Synapses.

In PHP, reduced current through postsynaptic GluRIIs (reduction in quantal size), due to either a genetic perturbation (the null mutation of the GluRIIA subunit, GluRIIA^−/−^) or pharmacological block of the GluRII pore with the wasp venom Philanthotoxin-433 (PhTox) or the small molecule GYKI 53655, is compensated for by an increase in the total amount of glutamate released by each AP (increase in quantal content or number of vesicles released) (20, 34). Because PHP occurs at Ib synapses but not Is synapses and is incomplete even at Ib synapses (32, 33), the compensation is only partial. This suggests that an additional homeostatic mechanism may be needed to ensure locomotor output. In search of such a mechanism, we first explored other synaptic perturbations that weaken MN-to-muscle synaptic transmission by turning from the postsynaptic perturbation to a presynaptic perturbation of glutamate release. We evaluated the effects of potential disruptions in release with optical quantal analysis of transmission by type Ib and Is inputs.

To perturb presynaptic transmitter release, we utilized a MN-specific Gal4 driver, OK6 (35), to conditionally drive expression in Ib and Is MNs of RNAis targeting genes that encode proteins of the transmitter release machinery. While the selected proteins are known to impact total quantal content (as measured electrophysiologically for total release), their impact on transmission specifically at Ib versus Is synapses has not been resolved. We tested: i) Cacophony (Cac), the voltage-gated Ca^2+^ channel that couples the AP to transmitter release, ii) Synaptotagmin 1 (Syt1), the Ca^2+^ sensor for AP-evoked release, iii) Rab3, the vesicular small GTPase, iv) the Rab3 interacting molecule (RIM), which clusters Ca^2+^ channel at the release site, v) the RIM-binding protein (Rbp) that connects release sites to the Bruchpilot active zone (AZ) scaffold, vi and vii) the regulators of synaptic vesicle priming Unc-13 and Unc-18, viii) the synaptic vesicle glutamate transporter (VGlut), ix) the AZ scaffolding protein Liprin-α, x) leukocyte antigen-related (LAR), a receptor tyrosine phosphatase, and xi) the Shaker potassium channel (Sh) (36–39).

**Fig. 1.**
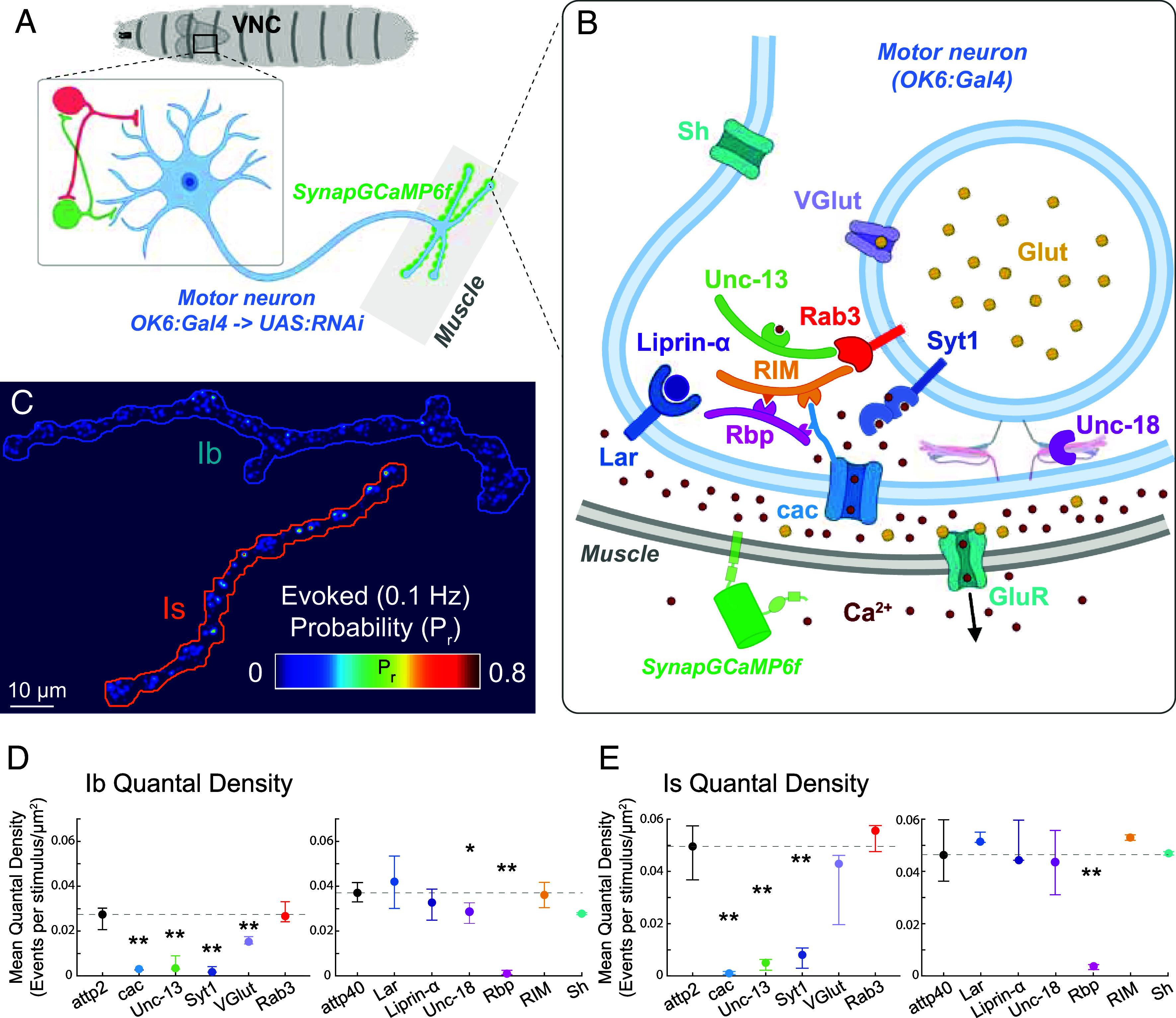
RNAi screen identifies presynaptic machinery knockdowns that weaken transmitter release by Ib and Is MNs. (*A*) Overview of RNAi screen. OK6 > Gal4 drives expression of RNAi in motor neurons, which receive excitatory and inhibitory input from upstream premotor neurons located in the VNC. SynapGCaMP6f imaging of postsynaptic Ca^2+^ rise due to influx via ionotropic GluRs in the muscle enables optical quantal analysis of transmission. (*B*) Diagram of the presynaptic active zone, postsynaptic muscle, presynaptic proteins targeted by the RNAis, and the postsynaptic GluR whose GluRIIA subunit is targeted by mutation. (*C*) Representative Pr heatmaps for Ib and Is MN synapses calculated from failure analysis during train of 100 stimuli at 0.1 Hz. (*D* and *E*) Mean quantal density (quantal content normalized to imaged NMJ area) for Ib synapses (*D*) and Is synapses (*E*) from control animals [attp2(empty) or attp40(empty)] and from animals with RNAi for one of 12 genes encoding indicated presynaptic proteins (Brp, Sh, cac, Syt1, Rab3, RIM, Rbp, Unc-13, Unc-18, VGlut, Liprin-α, and Lar) inserted in either the attp2 or attp40 site. Points are median values for larvae, and whiskers encompass the minimum and maximum values that are not outliers. Statistical comparisons Mann–Whitney test: *P < 0.05; **P < 0.01; ***P < 0.001.

Synaptic transmission was first imaged in the dissected preparation, where the brain and ventral nerve cord (VNC) are removed and nerve bundles containing Ib and Is MN axons are stimulated electrically. We visualized the Ca^2+^ component of the excitatory postsynaptic current (EPSC) through GluRII receptor (GluR) channels as an AP-evoked Ca^2+^ rise detected by SynapGCaMP6f, our genetically encoded reporter that localizes GCaMP6f to the postsynaptic density (Fig. 1 *A*–*C*) (32, 40–42). We measured quantal density, the number of evoked transmission events per stimulus per unit area of postsynaptic membrane (the optical equivalent of quantal content). As observed earlier (32, 43), in control animals, the quantal density of Is synapses was higher than that of Ib synapses (Fig. 1 *D* and *E*). In both Ib and Is synapses, quantal density was greatly reduced in the RNAis of Cac, Syt1, Rbp, and Unc-13 and reduced in Ib synapses in VGlut (Fig. 1 *D* and *E*). Lack of effect of the other RNAis could reflect ineffective knockdown or minimal contribution of that protein to release, so we did not interpret these negative results. We focused, instead, on three of the four MN RNAis that greatly reduced quantal release at both Ib and Is synapses: Cac, Rbp, and Unc-13. We confirmed that these RNAis did indeed knock down protein levels in the presynaptic nerve terminals (*SI Appendix*, Fig. S1). We note that perturbations of Rbp, Cac, and of the Cac auxiliary α2δ subunit have been shown to compromise PHP (19, 44, 45), so that the impact of the RNAis for Rbp and Cac could reflect a combined effect of their participation in release and PHP. The salient point for our study was that we now had in hand three presynaptic perturbations—RNAi knockdowns of Cac, Rbp Unc-13—and one postsynaptic perturbation—GluRIIA null mutant—that reduce the strength of transmission, whether or not PHP is engaged to compensate.

### Effect on Locomotion of Perturbation of Either the Presynaptic Release Machinery or Postsynaptic Glutamate Receptor.

Considering that PHP only partially compensates for postsynaptic weakening and that presynaptic reduction in AP-evoked release triggers no compensatory change in postsynaptic quantal size (32, 33), both classes of perturbation would be expected to result in a deficit in motor output. However, neither the postsynaptic perturbation, due to the GluRIIA^−/−^ mutant, nor the presynaptic RNAi knockdown of Cac, Rbp, or Unc-13 in type I MNs with the motor-neuron specific OK6-Gal4 (35, 46), prevented larvae from surviving to adult, suggesting that these disruptions are not too severe.

**Fig. 2.**
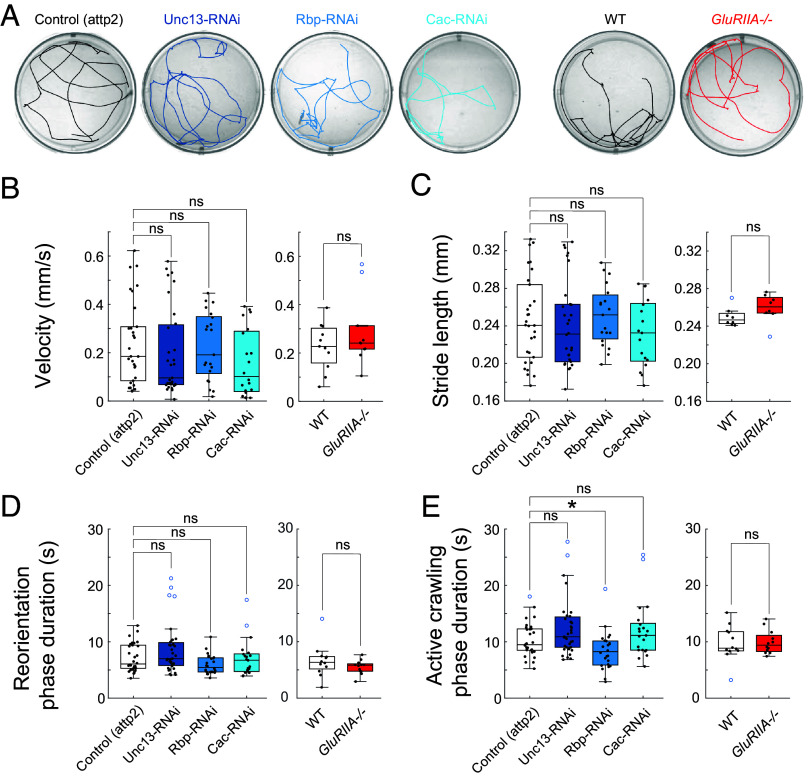
Crawling preserved despite impaired synaptic transmission. (*A*) Representative crawling tracks in 50 mm wells. (*B*–*E*) Locomotion kinematics of crawling of animals with MN-specific RNAi of Unc-13, Rbp, or Cac, compared to attp2(empty) control (*Left*), and GluRIIA^−/−^ compared to WT control (*Right*) (see *SI Appendix*, Fig. S2*F* for additional examples). (*B*) Mean velocity. (*C*) Mean stride length. (*D*) Mean duration in reorienting phase. (*E*) Mean duration of active crawling phase. (*B*–*E*) Points are average value for each larva. Box plots depict median, the lower and upper quartiles, any outliers (open circles, computed using the interquartile range), and whiskers encompass the minimum and maximum values that are not outliers. Statistical comparisons Mann–Whitney test: *P < 0.0.5; **P < 0.01; ***P < 0.001.

To examine the effect on motor behavior in detail, we tracked crawling activity of freely moving third-instar larvae on the bottom of an upright horizontal surface (Fig. 2*A*). As described earlier (47–49), larval locomotion alternated between two modes: active crawling and reorientation (Movie S1). The active crawling phase was characterized by peristaltic crawling that followed a relatively straight and persistent trajectory. Between periods of active crawling, there were phases of reorientation, where larvae ceased crawling, swept their heads from side to side, and then launched a new direction of forward crawling.

Strikingly, locomotion in each of the three presynaptic knockdowns and in the GluRIIA*^−/−^* mutant was similar to that seen in control animals, with no change in average velocity or average stride length (Fig. 2 *A*–*C* and *SI Appendix*, S2 *A* and *F*). We identified bouts of reorientation and measured their timing (*SI Appendix*, Fig. S2 *B* and *F*), but did not observe a change in the mean duration of reorientation bouts (Fig. 2*D*). The duration of the runs of continuous crawling on a single trajectory was also indistinguishable between control animals and animals with synaptic perturbations, except for a small (~14%) reduction in one of the four perturbations, the presynaptic Rbp RNAi (Fig. 2*E*). In other words, the synaptic perturbations had little or no effect on core aspects of locomotion. Only when we quantified larval agility—as measured by body curvature and turning angles between persistent crawling phases—did we observe a small behavioral impact of the synaptic perturbations. By fitting a spline down the midline of the larva we were able to calculate body curvature (*SI Appendix*, Fig. S2*C* and Movie S1). The curvature distributions were significantly shifted to smaller curvatures in all three of the presynaptic perturbations and in the postsynaptic perturbation (*SI Appendix*, Fig. S2*D*), but these effects were small in magnitude. We found a small but significant shift toward zero in the distribution of turning angles (angle between continuous trajectories) in the Unc-13 and Cac presynaptic RNAis and in the GluRIIA*^−/−^*animals, but not in the presynaptic Rbp RNAi (*SI Appendix*, Fig. S2*E*).

Thus, despite the large reduction in AP-evoked transmission in the presynaptic knockdowns of Cac, Rbp, and Unc-13, and incomplete compensation for the reduction in postsynaptic strength caused by mutation of the GluRIIA subunit, neuromuscular synapse-weakened larvae exhibited remarkably normal locomotor behavior, with only minor deficits in agility. We wondered whether a change in the MN firing pattern might compensate for reduced transmission by individual APs. To examine this, we next studied neural activity in the intact, behaving animal.

### Altered Neuromuscular Transmission Dynamics Compensates for Synaptic Weakening.

**Fig. 3.**
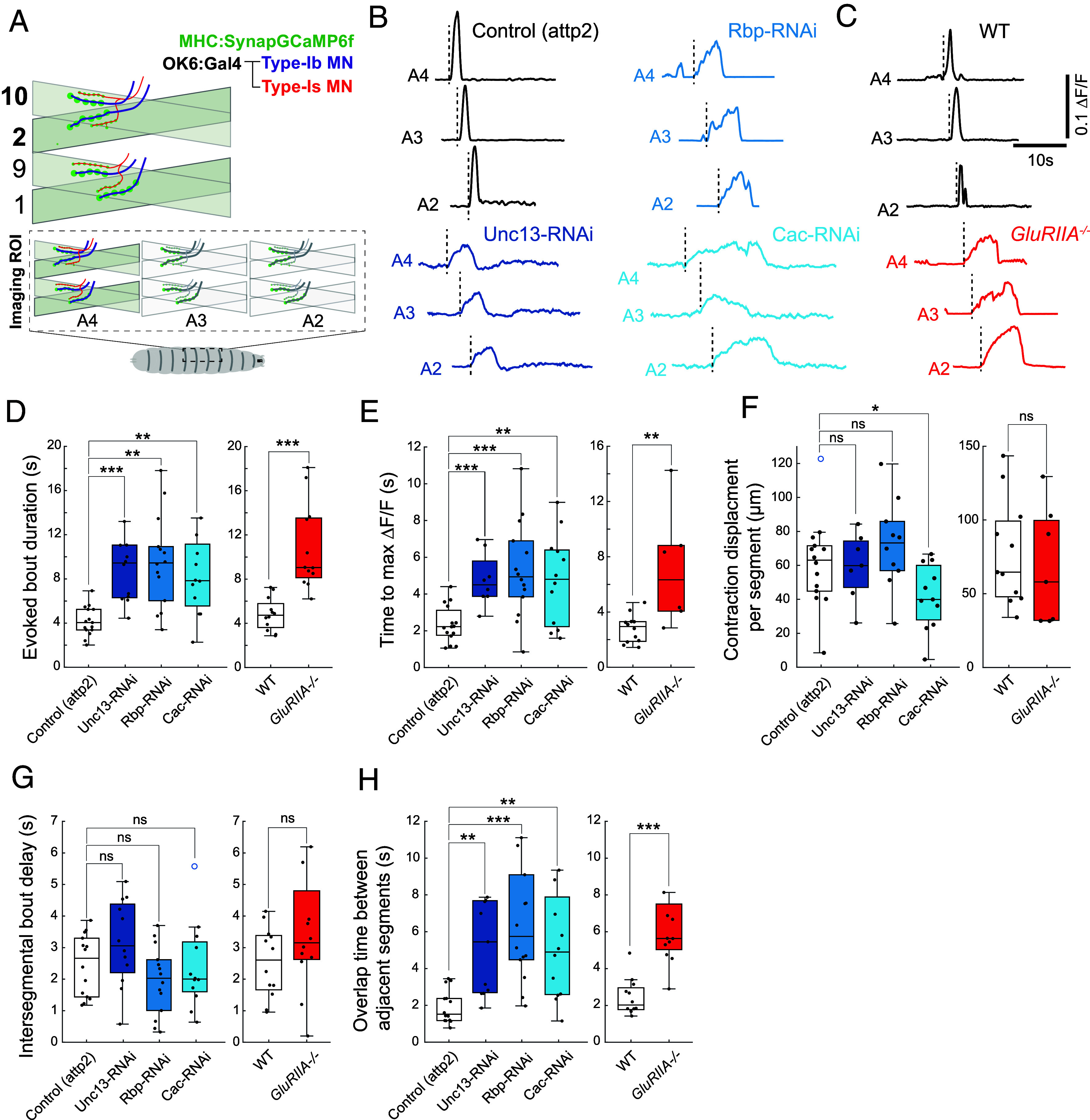
Pre- or postsynaptic weakening increases duration of synaptic transmission bouts during forward crawling waves. (*A*) Schematic of SynapGCaMP6f imaging of Ca^2+^ elevation due to influx through postsynaptic GluR channels during MN-to-muscle synaptic transmission in intact, restrained larvae within segments A4, A3, and A2. (*B* and *C*) Representative ΔF/F traces of SynapGCaMP6f in abdominal hemisegments A2, A3, and A4 during P → A peristaltic wave for Unc-13, Rbp, or Cac RNAis versus attp2(empty) control (*B*) and GluRIIA^−/−^ versus WT control (*C*). Start of bout marked by dashed vertical line. (*D*–*H*) Ib MN-to-muscle 2 or 10 synaptic transmission dynamics in animals with MN-specific RNAi of Unc-13, Rbp, or Cac, compared to attp2(empty) control (*Left*), and GluRIIA^−/−^ compared to WT control (*Right*). (*D*) Average duration of activity bout in A2-A4. (*E*) Time to maximal ΔF/F. (*F*) Contraction displacement per segment. (*G*) Intersegmental delay from A4 to A3 and A3 to A2. (*H*) Duration of overlap of bouts in A4 and A3, and in A3 and A2. Points are average value for each larva. Box plots depict median, the lower and upper quartiles, any outliers (open circles, computed using the interquartile range), and whiskers encompass the minimum and maximum values that are not outliers. Statistical comparisons Mann–Whitney test *P < 0.0.5; **P < 0.01; ***P < 0.001.

The *Drosophila* larva has 3 thoracic (T1-T3) and nine abdominal segments (A1-A9), most of which contain 30 bilateral body wall muscles. Each muscle is innervated by one of 26 different type Ib MNs, with a few muscles sharing a single Ib input, and the same 30 muscles innervated by two nonoverlapping Is MNs, each of which innervates multiple muscles (29). The posterior-to-anterior (P-to-A) propagation of peristaltic muscle contraction simultaneously contracts the left and right sides of each segment to propel the larva forward (50). To measure motor neuron input to the muscles while simultaneously observing forward-crawling peristaltic behavior, we turned to our restrained crawling setup, which consists of a linear chamber that restricts the larva to crawl in place while synaptic transmission is imaged in multiple segments (32). While confined to the chambers, the larvae exhibit activity patterns similar to those observed in freely crawling animals. These include forward- and backward-propagating peristaltic waves, overlapping contractions between adjacent segments, and dynamics consistent with the natural movement of freely crawling larvae (Fig. 3 and *SI Appendix*, Fig. S3) (47, 51, 52). The dynamics of synaptic transmission during crawling were measured by imaging of postsynaptic SynapGCaMP6f (Fig. 3*A* and *SI Appendix*, Fig. S3*A*).

Imaging from T1-A9 (Movie S2) revealed that ~95% of the time when neighboring abdominal segments A2-A4 participate in a P-to-A wave, this is part of a peristaltic wave that travels along the entire length of the larva. To maximize the signal-to-noise of SynapGCaMP6f transmission imaging during peristaltic wave behavior, we focused on NMJs in dorsal A2-A4 segments (*SI Appendix*, Fig. S3*A*). As reported previously (37), we observed that the dorsal muscle cells of each segment contract together during P-to-A waves (Movie S3). Because muscle cells cluster into four coactivated muscle groups, we were able to focus on one of these groups: Ib MNs innervating muscles 2 and 10 (Fig. 3*A*), which belong to the same peristaltic forward crawling group (37).

During propagation of P-to-A waves, each of the presynaptic release machinery knockdowns (RNAis of Unc-13, Rbp, and Cac), as well as GluRIIA^−/−^, had an altered temporal pattern of synaptic transmission: In all cases, bout duration nearly doubled (Fig. 3 *B*–*D*), and the bout rise phase slowed (Fig. 3 *B*, *C*, and *E*). The amount of contraction displacement was similar to that of control animals in all but the Cac RNAi (Fig. 3*F*). The speed of P-to-A wave propagation, which we measured as intersegmental bout delay, was also similar to control (Fig. 3*G*) and the delay between end of bouts, the peristaltic wave frequency and bout amplitude did not change consistently (*SI Appendix*, Fig. S3 *B*–*D*), but the presynaptic RNAi and GluRIIA^−/−^ mutant larvae had greater temporal overlap between synaptic transmission bursts in adjacent segments (Fig. 3*H*). These observations suggest that reduced glutamate release by each AP induces an increase in AP burst duration and burst overlap in successive segments and that these together maintain normal contraction.

### Activity Compensation Involves a Cell-Autonomous Increase in Type Ib MN Burst Duration.

**Fig. 4.**
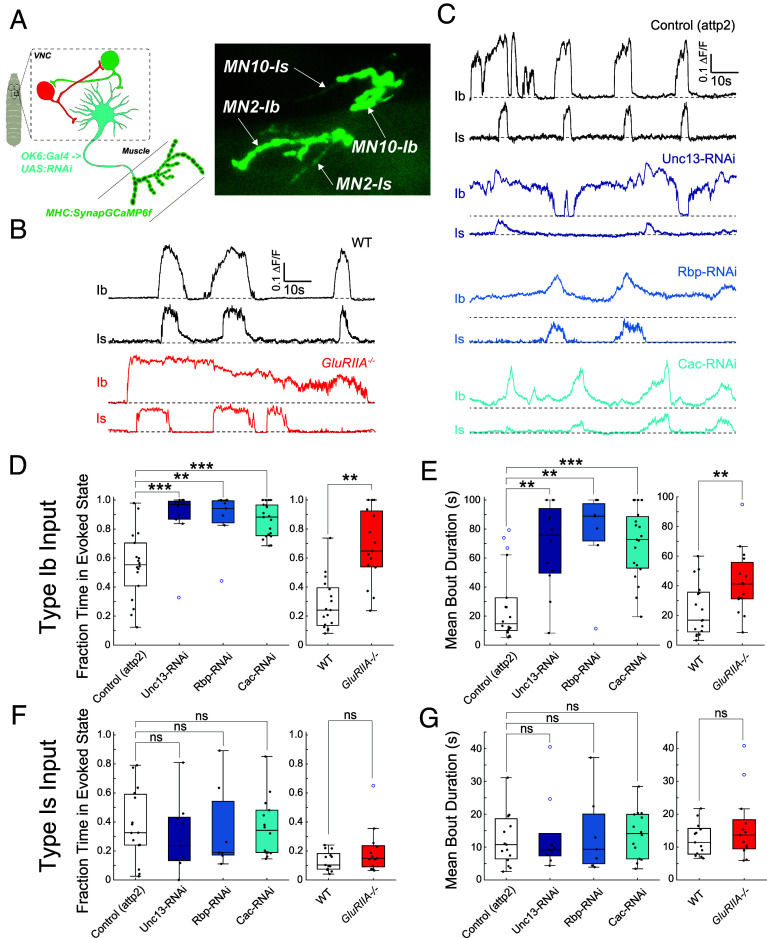
Increased MN burst activity is specific to Ib inputs. Dynamics of synaptic transmission imaged under high magnification in segment A1 to visualize both Ib and Is inputs. (*A*) Schematic of SynapGCaMP6f imaging (*Left*). Representative image of SynapGCaMP6 NMJ fluorescence at two Ib inputs (MN2 to muscle 2 and MN10 to muscle 10) and one Is input (to both muscles 2 and 10) in A1 hemisegment. (*B* and *C*) Representative SynapGCaMP6 ΔF/F traces for transmission by Ib and Is inputs in GluRIIA^−/−^ and WT control (*B*) and in presynaptic RNAis and their attp2(empty) control (*C*). Horizontal dashed lines represent the baseline. (*D*) Fraction time in evoked state for Ib input. (*E*) Mean ΔF/F bout durations for Ib input. (*F*) Fraction time in evoked state for Is input. (*G*) Mean ΔF/F bout durations for Is input. (*D*–*G*) Points are average value for each larva. Box plots depict median, the lower and upper quartiles, any outliers (open circles, computed using the interquartile range), and whiskers encompass the minimum and maximum values that are not outliers. Statistical comparisons Mann–Whitney test: *P < 0.0.5; **P < 0.01; ***P < 0.001.

Having observed activity compensation in Ib NMJs whose synaptic transmission was disrupted, we wondered whether such compensation also occurs at the Is NMJs onto the same muscles. To resolve the Is NMJs, which are smaller and whose SynapGCaMP6f signal is dimmer than the Ib NMJs, we increased magnification and focused on the NMJs of a single A1 segment including dorsal muscles 1, 2, 9, and 10 (Fig. 4*A* and Movie S4). Unlike our lower-magnification imaging, where we could observe peristaltic waves of locomotion traveling across multiple segments, our field of view for this set of experiments was confined to a single segment. As a result, we were unable to distinguish activity bouts associated with locomotory peristaltic waves from those restricted to the A1 and thoracic segments, such as those occurring during head-sweeping behaviors (46, 53). The integrated SynapGCaMP6f signal, which reflects total synaptic transmission, did not change consistently (preserved at control levels in two of the three presynaptic perturbations, decreased in the third, and increased in the GluRIIA^−/−^ mutant) (*SI Appendix*, Fig. S4*B*), while the degree of muscle contraction was preserved at control levels in all four cases of synapse perturbation (*SI Appendix*, Fig. S4*C*). A1 Ib NMJs approximately doubled fraction time active and bout duration in the Unc-13, Cac, and Rbp RNAi KDs, as well as in the GluRIIA null mutant (Fig. 4 *A*–*E*). Remarkably, in contrast to the changes seen in Ib synapses, no change was detected in the temporal pattern of synaptic transmission bouts in Is NMJs (Fig. 4 *F* and *G*).

**Fig. 5.**
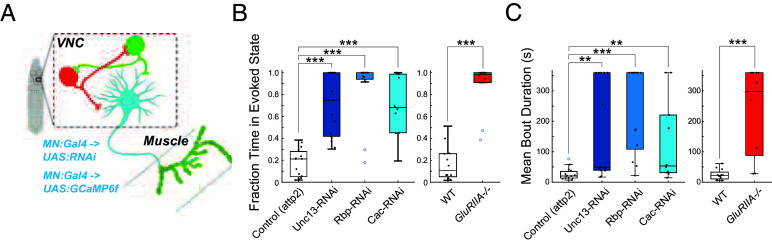
Effect of RNAis and GluRIIA^−/−^ on type Ib MN presynaptic activity. Type Ib MN activity measured with cytoplasmic GCaMP6f under high magnification in segment A1. (*A*) Schematic of imaging GCaMP6f in MNs. (*B* and *C*) Fraction time in evoked state (*B*) and mean bout duration (*C*) for presynaptic RNAis compared to attp2(empty) control and GluRIIA^−/−^ compared to WT control. Box plots depict median, the lower and upper quartiles, any outliers (open circles, computed using the interquartile range), and whiskers encompass the minimum and maximum values that are not outliers. Statistical comparisons Mann–Whitney test: *P < 0.05; **P < 0.01; ***P < 0.001.

Having observed increases in the duration and fraction active time of synaptic transmission bouts from type Ib MNs, we sought to directly measure the dynamics of MN firing. To do this, we imaged cytosolic GCaMP6f in the *presynaptic* type Ib terminal axons. These recordings showed that each of the animals with weakened neuromuscular synaptic transmission increase in both fraction time active and burst duration in Ib MNs (Fig. 5), consistent with the effects on synaptic Ib MN transmission dynamics.

We recently found that type I MN-selective knockdown of either Unc-13 or Rbp reduces expression of a set of voltage-gated K^+^ (K_v_) channels and auxiliary subunits that boost K_v_ function (54). Together with our findings here, this suggests that reduced K_v_ function may compensate for weakened neuromuscular transmission by increasing excitability, thereby increasing the duration of type Ib MN activity bouts. In that study, three K_v_s were found to decrease in expression: the A-type Shaker K_v_1 channel, the delayed rectifier Shab K_v_2 channel, and the HERG-like Elk channel (54). Mutants of Shaker and Shab have been shown to increase the duration of type I MN locomotory bursts in a semi-intact larval neuromuscular preparation (55). However, since the mutants affect neurons throughout the nervous system, their impact could be more substantial than if their disruption was limited to MNs.

To test the hypothesis that a homeostatic reduction in MN expression of these K_v_s contributes to the increased duration of locomotory type Ib MN transmission bouts, we directly knocked down two of these K_v_s exclusively in type I MNs. We find that knockdown of either Shab (OK6-Gal4; UAS*-Shab^RNAi^*) or Shaker (OK6-Gal4; UAS*-Sh^RNAi^*) has a similar effect to that of synaptic weakening: increasing type Ib MN synaptic transmission bout duration and temporal overlap between adjacent segments, with no change in intersegmental bout delay (*SI Appendix*, Fig. S3 *E*–*G*). Together, these observations suggest that increased type Ib MN firing due to a cell-autonomous decrease in K_v_ function contributes to compensation for weakening of neuromuscular transmission.

### Altered Activity in a Central Premotor Input Associated with MN Firing Homeostasis.

The large increase that we observed in MN burst duration when synaptic transmission from MN to muscle is weakened led us to wonder whether additional mechanisms, beyond increased type Ib MN excitability, contribute to increased type I MN activity. We considered that there may also be changes elsewhere in the locomotory circuit. We therefore turned our attention upstream of the MNs to the premotor neurons (PMNs). Because we observed firing homeostasis in four type Ib MNs that innervate muscles 1, 2, 9, and 10 (MN1-Ib, MN2-Ib, MN9-Ib, and MN10-Ib), but not in the single type Is MN (MNISN) that innervates the same muscles, we focused on PMNs that innervate those type Ib MNs and not that type Is MN. These included three sets of PMNs: period-positive median segmental interneurons (PMSIs, also known as A02 cells), A18a and A31b cells (37, 56, 57). Activity in PMSIs has been shown to reduce type I MN burst duration during locomotory waves (56), making them particularly interesting candidates. Moreover, among these PMN cell types, only the PMSIs have a LexA driver (R70C01-LexA) that could be combined with the UAS-RNAi, which we use to knock down the presynaptic release machinery. R70C01-LexA drives expression in both A02h, which innervates all four of the type Ib MNs that we imaged, but not MNISN and A02e, which innervates one of the four type Ib MNs (MN10-Ib) but not MNISN (37, 57).

**Fig. 6.**
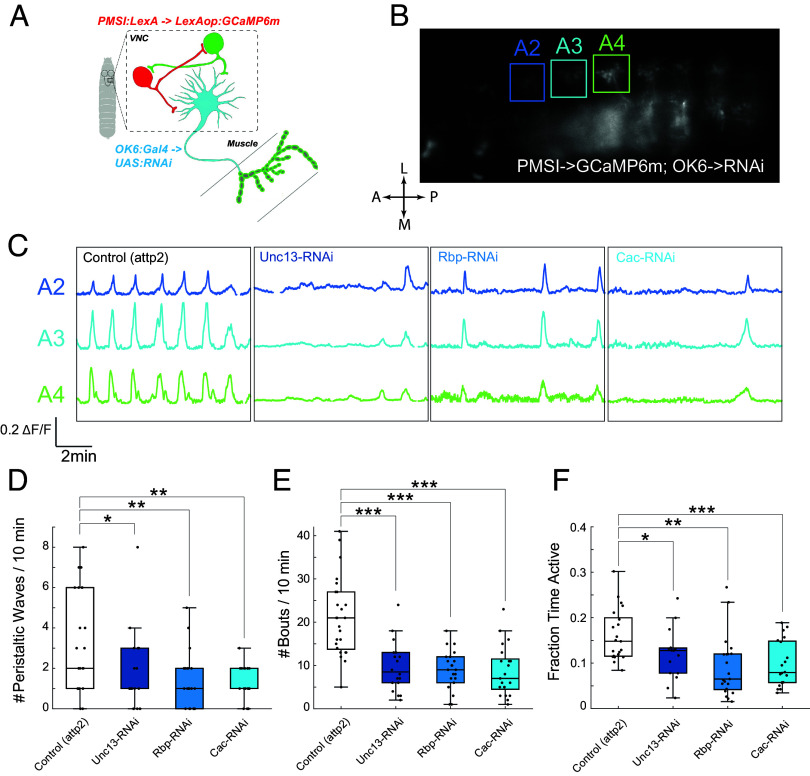
Weakening of MN to muscle synapse decreases activity of PMSI inhibitory premotor neurons. (*A* and *B*) Schematic (*A*) and image of cytosolic GCaMP6m (R70C01-LexA) activity during a P → A peristaltic wave in PMSIs (*B*). (*C*) Representative GCaMP6f ΔF/F traces in PMSIs of segments A2, A3, and A4 traces in animals with MN-specific RNAi for Unc-13, Rbp, or Cac, compared to attp2(empty) control. (*D*–*F*) Number of peristaltic waves per 10 min (*D*), mean number of ΔF/F activity bouts per 10 min (*E*), and fraction time active (*F*). Box plots depict median, the lower and upper quartiles, any outliers (open circles, computed using the interquartile range), and whiskers encompass the minimum and maximum values that are not outliers. Statistical comparisons Mann–Whitney test: *P < 0.0.5; **P < 0.01; ***P < 0.001.

We expressed cytosolic GCaMP6m in PMSIs using R70C01-LexA, while we knocked down Unc-13, Rbp, or Cac with UAS-RNAi exclusively in type I MNs using OK6-Gal4, as before (Fig. 6*A*). We imaged activity in PMSI processes in the VNC across the abdominal segments, where the PMSI processes are clearly restricted to single segments (Fig. 6*B*). In PMSIs of control animals, we observed regular bouts of inhibitory activity that were uniform in amplitude and duration and traveled in both the P→A and A→P directions (Fig. 6 *C*, *Left* and Movie S5). In each of the release machinery knockdowns, there was a dramatic decrease in PMSI activity, including reduced peristaltic wave frequency, bout frequency, and fraction active time (Fig. 6 *C*–*F*). While the frequency of these events was altered, their duration and magnitude were not (*SI Appendix*, Fig. S5 *A* and *B*), and there was no effect on the intersegmental delay between the onset of bouts during peristaltic waves (*SI Appendix*, Fig. S5*C*). These observations suggest that increased firing of type Ib MNs in animals with weakened MN to muscle synaptic transmission involves an upstream circuit adjustment that decreases inhibitory drive to Ib MNs.

### Timing of Homeostatic Firing Adjustment in Type Ib MNs.

**Fig. 7.**
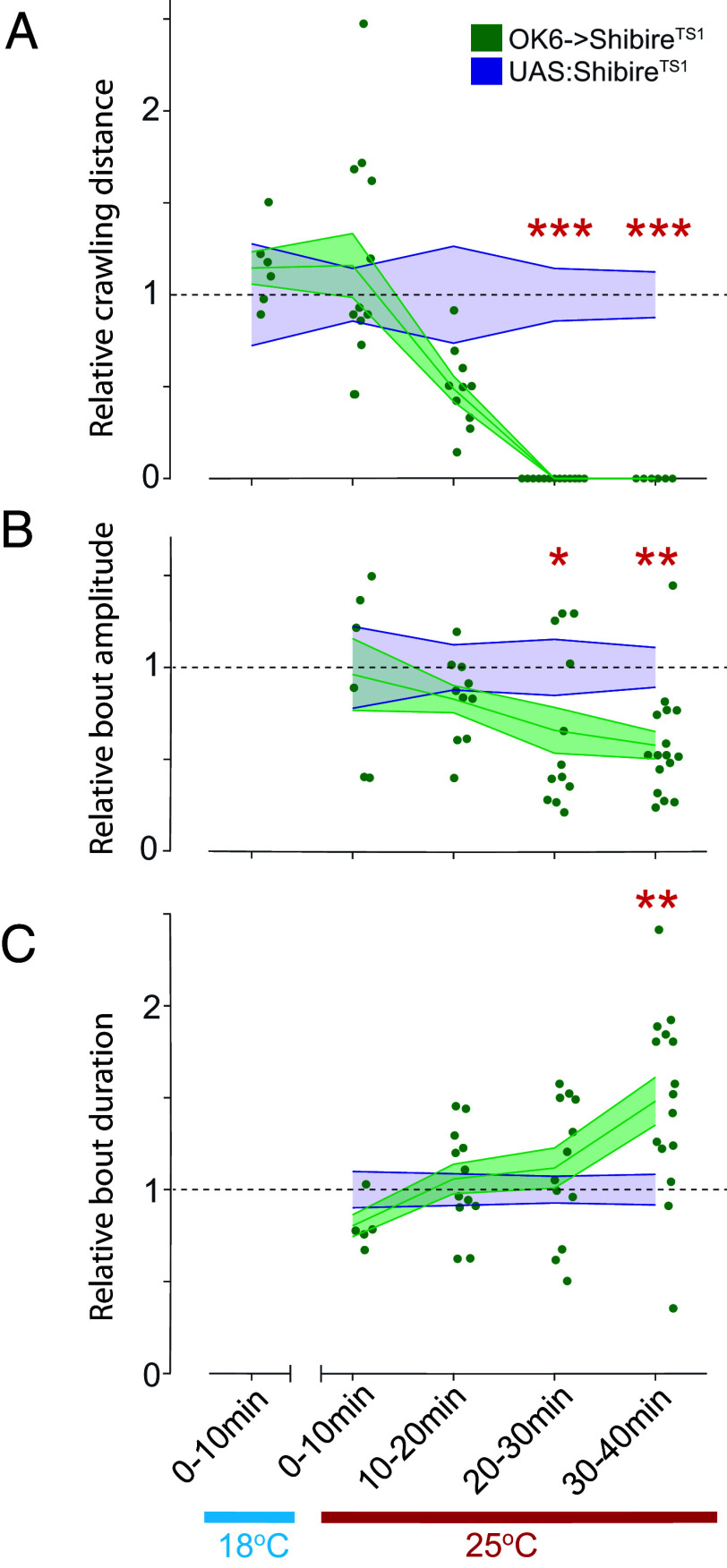
Time course of Ib synaptic transmission bout duration increases following Shibire^TS1^-induced decrease in synaptic transmission. (*A*) Change in relative crawling distance following elevation to restrictive temperature in OK6->Shibire^TS1^. (*B* and *C*) Change in relative SynapGCaMP6f ΔF/F bout amplitude (*B*) and duration (*C*) following elevation to restrictive temperature. (*A*–*C*) Comparison of OK6->Shibire^TS1^ to UAS:Shibire^TS1^ control. Points are average value for each larva. Error clouds represent SEM. Statistical comparisons Mann–Whitney test: *P < 0.05; **P < 0.01; ***P < 0.001.

PHP at the *Drosophila* larval NMJ begins to boost transmitter release within minutes after block of postsynaptic GluRII by PhTox (19). We wondered whether the increase in Ib MN activity, which we observe in synaptic weakening, also occurs quickly. Since PhTox cannot be readily introduced into the intact larva and reduced expression of GluRIIA occurs slowly following a genetic manipulation, we turned to the temperature-sensitive *Drosophila* dynamin mutant, *shibire*^ts1^ (*shi*^ts1^), which reduces transmitter release at the restrictive temperature within minutes, due to block of vesicle recycling (58). We expressed UAS-*shi*^ts1^ in type I MNs under OK6-Gal4. Initially, we held larvae at a permissive temperature (18 °C) and then switched to a restrictive temperature (26 °C) and imaged synaptic transmission in segments A2-A4 during P → A peristaltic waves over successive 10 min bins, following transition to the restrictive temperature. We compared *shi*^ts1^-expressing animals (UAS-*shi*^ts1^; OK6-Gal4) to controls (UAS-*shi*^ts1^) that lacked the Gal4 driver (Fig. 7). At the restrictive temperature, locomotion dropped in the *shi*^ts1^ expressing larvae compared to control in the 10 to 20 min bin and halted in and beyond the 20 to 30 min bin (Fig. 7*A*), while synaptic transmission bout amplitude, measured with SyanpGCaMP6f, dropped significantly starting in the 20 to 30 min bin (Fig. 7*B*). Synaptic transmission bout duration increased in the 30 to 40 min bin (Fig. 7*C*). Thus, the homeostatic change in type Ib MN activity pattern occurs 10 to 20 min following acute presynaptic reduction in glutamate release.

## Discussion

Homeostatic mechanisms in the nervous system maintain a balance between excitation and inhibition and ensure circuit throughput by adjusting synaptic transmission and excitability following activity-dependent plasticity changes and pathological perturbations (5, 13, 20, 59). PHP in the *Drosophila* larval neuromuscular junction compensates for postsynaptic reduction in sensitivity to transmitter by increasing transmitter release via a retrograde signaling system (18–20, 31). However, at physiological Ca^2+^, PHP in response to the GluRIIA^−/−^ mutation only occurs in one of the two excitatory glutamatergic inputs to muscle–the type Ib MN–and even within the Ib input, PHP only partially compensates for the reduction in the amplitude of the postsynaptic quantal response (32). [Note that this differs from the situation with PhTox block of GluRII where compensation is complete (33)]. We find that larvae with either a presynaptic reduction in glutamate release or postsynaptic decrease of GluRII function *increase* the duration of locomotory MN bursts and the total fraction of time active during both locomotory waves and nonlocomotory movement. In contrast to the all-or-none contraction dynamics of skeletal muscle in vertebrates that produce a single, maximal contraction, the graded response of larval body wall muscle to varying MN firing frequencies allows for fine-tuning of muscle contraction. Larvae with weakened synapses, therefore, may achieve the same degree of contractile force as wildtype larvae by increasing MN firing and thereby increasing the summation of signal to the muscle. This adjustment occurs in the tonic type MN1-Ib, MN2-Ib, MN9-Ib, and MN10-Ib, but not in the type Is MNISN that innervates the same muscles. Crawling velocity has been shown to be regulated by peristaltic wave frequency rather than stride length (51). In accord with this, we observe that peristalsis wave speed and frequency are preserved in both the pre- and postsynaptically weakened larvae, consistent with the observed maintenance of normal crawling velocity. The increased activity is accompanied by an increase in the amount of time that neighboring segments are simultaneously active, which may help to generate the same contractile displacement that individual segments normally exert. Remarkably, the type Ib MN increase in synaptic transmission bout duration offsets the single AP-triggered reduction in postsynaptic Ca^2+^ influx through the GluR channels, suggesting that the homeostatic mechanism operates to maintain a setpoint of integrated intracellular Ca^2+^ at the postsynaptic density.

Two parallel homeostatic mechanisms appear to contribute to the compensatory increase in MN activity and transmitter release that are triggered by synaptic weakening. The first mechanism is a cell-autonomous change in the MNs that makes them more excitable. Griffith and Marder (60) showed that a mutation in the Eag K_v_ channel, which reduces glutamate release, also increases type Ib MN excitability, although this was complicated by the fact that Eag itself regulates excitability. We found recently that presynaptic weakening due to knockdown of Unc-13 or Rbp in type I MNs reduces expression of a type I MN K_v_ gene module, consisting of three genes encoding K_v_ channel pore-forming subunits [Shaker (K_v_1), Shab (K_v_2), and Elk (K_v_10)] and three genes encoding K_v_ positive modulators [Hyperkinetic (Hk) and Quiver/Sleepless (Qvr/Sss), which boost Shaker function, and SKIP, which boosts Shal (K_v_4) function] (54). We find that direct knockdown of Shab or Shaker exclusively in type I MNs has the same effect as synaptic weakening: increase type Ib MN burst duration. These results suggest that decreased K_v_ function in type Ib MNs contributes to the compensatory elongation of activity bursts in type Ib MNs.

**Fig. 8.**
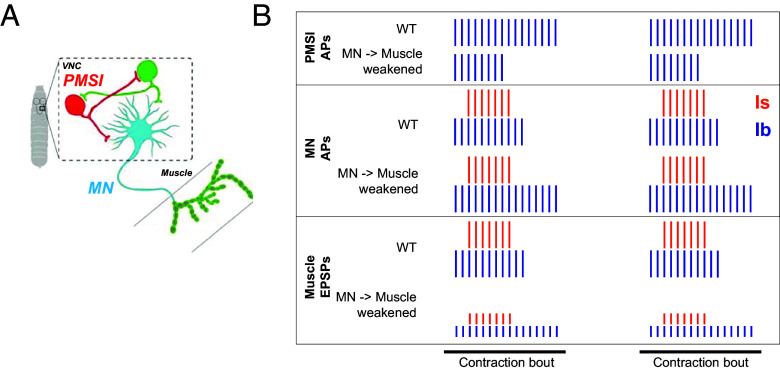
Model of motor neuron firing homeostasis. Reduced central inhibitory drive and increased excitability increase motor neuron firing to compensate for weakened motor neuron to muscle synaptic transmission. (*A*) Schematic of inhibitory premotor neurons synapsing onto type Ib MNs (inhibitory PMSI premotor neuron, red; excitatory premotor neuron, green). (*B*) Activity during locomotion of PMSIs (*Top*), type I MNs (*Middle*), and muscle (*Bottom*) under normal neuromuscular transmission (WT) and with presynaptic or postsynaptic perturbation that reduces synapse strength (MN→Muscle weakened). In compensation for neuromuscular transmission weakening, PMSIs fire less (reducing inhibition of type Ib MNs, but not type Is MNs, which they do not innervate). Type Ib MNs also increase in intrinsic excitability resulting in prolonged locomotory bursts that preserve near normal contraction and locomotion.

The second firing homeostasis mechanism is a change in the activity of PMSIs, which provide central inhibitory premotor neuron input to type Ib MNs, but not the type Is MN. We observe that, in synaptically weakened animals, locomotion-associated burst activity is decreased in PMSIs. These results agree with earlier work that showed that optogenetic stimulation that increases the activity of PMSIs has the opposite effect of shortening MN burst duration (56). Interestingly, PMSIs innervate MN1-Ib, MN2-Ib, MN9-Ib, and MN10-Ib, but not in the type Is MNISN that converges on the same muscles (37). Thus, weakening of synaptic transmission from type I MNs to muscle triggers a two-pronged change that increases type Ib MN firing: a cell-autonomous increase in type Ib MN excitability and a circuit adjustment that reduces inhibitory input to type Ib MNs (Fig. 8). Firing homeostasis and PHP are complementary and multiplicative: Firing homeostasis increases the number of APs and PHP increases glutamate release by each AP.

The locus and mechanism through which deficits in glutamatergic transmission and muscle contraction are detected by the larval nervous system, as well as how this signal is relayed upstream in the network to alter excitability and firing patterns, remain to be determined. We propose two potential pathways that might function independently or synergistically: 1) a two-step retrograde synaptic signaling mechanism, involving retrograde synaptic signaling from muscle to MN, and subsequently from MN to PMN; or 2) sensory feedback from the periphery to the central nervous system. In the case of retrograde signaling, it is plausible that the Sema2b to PlexB molecular mechanism that has been implicated in presynaptic homeostatic plasticity (31) could participate and, thereby, provide a coordinated control of transmitter release per AP and the number of APs. Alternatively, sensory neurons within the larval proprioceptive system may detect deficits in muscle contraction and drive compensatory changes in excitability of PMNs and MNs. Along these lines, multidendritic sensory neurons have been shown to be critical to control of MN firing duration in that their inactivation increases burst duration (61).

Type Ib inputs have low-release probability synapses that tend to facilitate, and their activity ramps up and down slowly, whereas type Is inputs have high-release probability synapses that tend to depress, and their activity turns on and off abruptly so that they burst only during the maximal contraction phase (32, 62, 63). The input selectivity of both PHP and firing homeostasis to Ib MNs allows Is MNs to maintain constancy, ensuring stable locomotor wave dynamics. Meanwhile, the adaptable input from Ib MNs provides plasticity to accommodate changing conditions. Together, these mechanisms achieve a balance of stability and flexibility, supporting robust locomotor behavior.

## Experimental Methods

### *Drosophila*.

Transgenic flies were generated through standard germline transformation via embryo injection (BestGene, Chino Hills, CA). 24B-Gal4 (64), GluRIIASP16, and Df(2L)clh4 (18) flies were from Corey Goodman (UC Berkeley). UAS-Shibire^TS1^ (65) was from Kristen Scott (UC Berkeley). OK6-Gal4 (#64199), R70C01-LexA (#54927), UAS-GCaMP6f, UAS-GCaMP6m, LexAop-GCaMP6m, UAS-Dicer2, and UAS-RNAi (TRiP) lines using either the attp2 or attp40 docking sites were from Bloomington *Drosophila* Stock Center (Bloomington, IN). Flies were grown on a standard corn meal and molasses diet at 25 °C, except those with RNAis, which were raised at 28 °C, or those used in Shibire^TS1^ experiments, which were raised at 18 °C. Experiments were conducted using both male and female wandering third instar larvae. Only actively crawling larvae were used for experiments.

The following genotypes were used:

Control (attp2) (UAS-Dicer2;OK6-Gal4/+; SynapGCaMP6f/attp2 (empty)),

Cac (UAS-Dicer2;OK6-Gal4/+;SynapGCaMP6f/UAS-Cac-RNAi (#27244),

Unc-13 (UAS-Dicer2;OK6-Gal4/+;SynapGCaMP6f/UAS-Unc13-RNAi (#29548),

Syt1 (UAS-Dicer2;OK6-Gal4/+;SynapGCaMP6f/UAS- Syt1-RNAi (#28508),

vGlut (UAS-Dicer2;OK6-Gal4/+;SynapGCaMP6f/UAS-vGlut-RNAi (#27538),

rab3 (UAS-Dicer2;OK6-Gal4/+;SynapGCaMP6f/UAS-rab3-RNAi (#25953),

Control (attp40) (UAS-Dicer2;OK6-Gal4/attp40 (empty); SynapGCaMP6f/+),

LAR (UAS-Dicer2;OK6-Gal4/UAS-LAR-RNAi (#40938); SynapGCaMP6f/+),

Liprin-α (UAS-Dicer2;OK6-Gal4/UAS-Liprin-α-RNAi (#28301); SynapGCaMP6f/+),

Unc-18 (UAS-Dicer2;OK6-Gal4/UAS-Unc18-RNAi (#51925); SynapGCaMP6f/+),

RBP in attp40 (UAS-Dicer2;OK6-Gal4/UAS-RBP-RNAi (#54828);SynapGCaMP6f/+),

RBP in attp2 (UAS-Dicer2;OK6-Gal4/+;SynapGCaMP6f/UAS-RBP-RNAi (#29312)),

RIM (UAS-Dicer2;OK6-Gal4/UAS-RIM-RNAi (#55741); SynapGCaMP6f/+),

RBP-3xFLAG (w1118; OK6-Gal4/UAS-Dicer2; attP2 (empty) /RBP-3xFLAG),

RBP-RNAi (w1118; OK6-Gal4/UAS-Dicer2; UAS-RBP RNAi/RBP-3xFLAG).

Sh (UAS-Dicer2;OK6-Gal4/UAS-Sh-RNAi (#55347); SynapGCaMP6f/+),

Control (attp2 for Shab-RNAi comparison) (w1118;OK6-Gal4/UAS-Dicer2; SynapGCaMP6f/attp2),

Shab (w1118;OK6-Gal4/UAS-Dicer2;SynapGCaMP6f/ UAS-Shab-RNAi (#55682)),

Sh (w1118;OK6-Gal4/UAS-Dicer2;SynapGCaMP6f/ UAS-Sh-RNAi (#31680),

wild-type control (w1118;+;+), wild-type SynapGCaMP6f (WT; w1118;+/+;SynapGCaMP6f/+),

GluRIIA null (GluRIIASP16/Df(2L)clh4;SynapGCaMP6f/+),

OK6>Shibire^TS1^ (w1118;OK6-Gal4/+;SynapGCaMP6f/UAS- Shibire^TS1^),

UAS-Shibire^TS1^ (w1118;+;SynapGCaMP6f/UAS- Shibire^TS1^,

PMSI Control (UAS-Dicer2; R70C01-LexA/OK6-Gal4; attp2 (empty)/LexAop-GCaMP6m),

PMSI Unc13-RNAi (UAS-Dicer2; R70C01-LexA/OK6-Gal4;UAS-Unc13-RNAi/LexAop-GCaMP6m),

PMSI RBP-RNAi (UAS-Dicer2; R70C01-LexA/OK6-Gal4;UAS-RBP-RNAi/LexAop-GCaMP6m),

PMSI Cac-RNAi (UAS-Dicer2; R70C01-LexA/OK6-Gal4;UAS-Cac-RNAi/LexAop-GCaMP6m).

### Larval Locomotion Assay.

For the larval locomotion assay, a Corning six-well plate was used containing 35 mm diameter wells. Each well was filled with 2% agarose gel, leaving an ~3 mm gap between the gel surface and the plate lid to restrict larval movement in the z-direction. The agarose was sufficiently dense to keep the larvae crawling on the surface rather than burrowing into the gel. Each well contained a single larva to enable individual tracking. The plate was backlit with an IR LED array within a chamber containing diffuse 60 W white light. Recordings were performed using a CCD camera (UniBrain, Fire-i 780b) for 10-min durations at rate of 10 frames per second. The temperature of the imaging chamber was maintained at 21 °C.

### SynapGCaMP6f Optical Quantal Imaging.

Optical quantal imaging of SynapGCaMP6f was performed as previously described (32). Briefly, third instar larvae were initially dissected in HL3 solution containing (in mM): 70 NaCl, 5 KCl, 0.45 CaCl_2_·2H_2_O, 20 MgCl_2_·6H_2_O, 10 NaHCO_3_, 5 trehalose, 115 sucrose, and 5 HEPES, with pH adjusted to 7.2. Following brain removal, larval fillets were imaged in HL3 solution containing 1.5 mM Ca2+ and 25 mM Mg2+. Fluorescence imaging was performed using a Vivo Spinning Disk Confocal microscope equipped with a 63× water immersion objective, a 488 nm laser, a Yokogawa spinning disk, a GFP filter, and an EMCCD camera. Recordings were conducted on ventral longitudinal abdominal muscle 4 at segments A2-A5, selecting fields of view to capture both Ib and Is terminals within a single focal plane.

Nerve stimulation was applied via a suction electrode connected to a Stimulus Isolation Unit, with intensity adjusted to recruit both axons, verified during imaging. Stimulation and imaging were synchronized using MATLAB scripts, triggering imaging episodes in SlideBook software. Episodic stimulation and imaging involved acquiring a series of 10 images (50 ms exposures) per episode. Imaging involved episodic acquisition of 10-image sequences (50 ms exposures), including 3 to 4 baseline frames before nerve stimulation. A total of 200 stimulus trials at 0.1 Hz ensured accurate analysis of release probability while minimizing statistical fluctuations.

### Generation of 3xFLAG Epitope–Tagged Rbp.

For measurements for Rbp, the control and experimental lines followed equivalent experimental design but with the inclusion of a 3xFLAG epitope–tagged RBP on the third chromosome (generated by WellGenetics). Briefly, gRNA plasmid and donor plasmid were injected into a vasa-Cas9 line to produce germline integration validated in the subsequent generation by expression of the 3xP3-DsRED fluorescent eye reporter, later excised by removal of the flox cassettes bounding the reporter (66). Validation of the CRISPR line was done by PCR and sequencing, as well as antibody staining to indicate successful localization of the tagged protein to synapses at motor neuron terminals.

### Antibodies and Immunofluorescence.

Across all three experiments to validate RNAi efficacy of Unc-13RNAi, Cac-RNAi, and Rbp-RNAi for their respective targets consisting of the knockdown of Unc-13A, Cac, and Rbp, respectively, control and RNAi larvae (6 for each; 12 total per experimental condition) were dissected and then stained in independent 5 mL Eppendorf tubes. Larvae were fixed in room temperature Bouin’s fixative for 5 min, permeabilized in PBS with 0.1% Triton X100 (PBT), and kept at 4*C while for 2 h while ice-cold PBT was exchanged every 30 min. Preparations were then blocked in PBS with 0.1% Triton X100, 2.5% normal goat serum, and 0.02% sodium azide (PBN). All antibody incubations were performed in PBN and all washes were performed in PBT with continuous nutation. Across all experiments, mouse anti-Brp (nc82; Developmental Studies Hybridoma Bank, Iowa City, IA) was used at 1:1,000 to identify synaptic active zones, while goat anti-HRP AlexaFluor 647 (Jackson ImmunoResearch, 123-605-021) was used at 1:500 to indicate motor neuron termini. Preceding each addition of antibodies, 3 × 10 min PBT washes followed by 30 min PBN blocking were conducted at room temperature. Rabbit anti-Cac antibody was used at 1:500 for 12 h at 4*C (Morton lab). Subsequently, for reduction of erroneous background staining, an additional 8-h wash in PBT at 4*C was conducted with continuous nutation. For secondary labeling of the Cac primary antibody, anti-Brp and anti-rabbit Biotin F(ab′)2 (Jackson 111-066-144) at 1:1,000 for 12 h at 4*C, followed by Streptavidin-555 at 1:500 (Thermo Fisher, S32357) for 2 h at room temperature. Rabbit anti-Unc13A was used at 1:1,000 (Sigrist Lab). Donkey anti-FLAG was used at 1:1,000 (Novus NBP1-06712). Secondary antibodies were incubated for 2 h at room temperature with nutation at 1:1,000 concentration; Donkey anti-Mouse IgG (H + L) Highly Cross-Adsorbed AlexaFluorPlus 488 (ThermoFisher, A32766), F(ab′)2-Goat anti-Rabbit IgG (H + L) Cross-Adsorbed Alexa Fluor Plus 555 (ThermoFisher, A48283), and donkey anti-rat AlexaFluor 555 plus (ThermoFisher, A48270).

### Airyscan Imaging and Analysis.

Following antibody incubations and washes, larval fillet buffers were exchanged with Vectashield Vibrance (H-1700; Vector Laboratories, Burlingame, CA) and mounted in 42 μL of Vectashield Vibrance beneath a high precision coverslip with square dimensions of 22 × 22 mm, a depth of 170 ± 5 μm, and refractive index of 1.5255 (Deckgläser). Airyscan2 imaging was performed on a Zeiss LSM 980 microscope. Samples were imaged with a ×63 oil immersion objective (NA 1.4, DIC; Zeiss) using Zen software (Zeiss Zen Blue 3.9). All imaging data across all three RNAi and respective control conditions were acquired using identical settings and displayed with equivalent contrast. Briefly, each imaging volume was acquired with an additional magnification of 4× with a 5 AU pinhole, 0.66 µs pixel dwell times, line scanned bidirectionally with no line averaging, an x-y dimension of 792 by 792 px yielding 43 by 43 nm/px, and axial z spacing of 150 nm. Each of the three-channel volumes (anti-Hrp Cy3, the indicator to the three different proteins of measurement [anti-Unc13A rabbit/anti-rabbit Alexa555+, anti-FLAG rat/anti-rat Alexa555+, anti-Cac rabbit/anti-rabbit biotin/Streptavidin Alexa555], and anti-Brp/anti-mouse Alexa 488+) were scanned sequentially. Respective laser powers and gain were 0.1% at 925 V, 0.25% at 900 V, and 0.1% at 875 V. Here, anti-HRP Alexa Fluor 647 was excited with 639 nm and LP570 filter, Alexa Fluor 555 was excited with 561 nm and SP615, and anti-mouse Alexa Fluor 488 was excited with 488 nm and SP550 filter. Subsequent deconvolution processing was conducted using Huygen’s Deconvolution (Scientific Volume Imaging, Hilversum, Netherlands); Good’s MLE was applied to each volume for a maximum of 10 iterations, and the deconvolved images were utilized to calculate a chromatic aberration correction (x, y, z) that was applied uniformly to all acquisitions. To display synaptic sites at terminal boutons, images were imported into Fiji ImageJ (NIH), and the maximum intensity frame for the Brp channel was selected. The contrast selected for the 488 channel for Brp was from 0:30,000; the 555 channel was from 0:15,000. To determine the full width half maxima and peak fluorescence intensity of individual synapses, line profiles were measured in Fiji at the maximum intensity position for Brp on a synapse-by-synapse basis. The line profiles were drawn at roughly 1 μm in length and placed to bisect both Brp and the synaptic protein of interest along their central axis. A custom Python script was used to fit a Gaussian function for each channel, and data were centered for the nearest position to the Gaussian peak (as true peak values are sensitive to local maxima). Mean and SEM values were calculated for each position within ±0.4 μm of the center at 42.5 nm intervals. A subsequent Gaussian function was fit to the mean values, and the full width half maximum was calculated using 2.355*sigma (representing the SD of a normal distribution).

### In Vivo Intact Larval SynapGCaMP6f Imaging.

In vivo imaging of intact larvae was conducted as previously described (32). For in vivo imaging, third instar larvae were placed in custom-made gas-permeable poly(dimethylsiloxane) (PDMS). For imaging, larvae were mounted in the PDMS chamber, which had an internal depth of 500 μm and width of 1 mm. The larvae were oriented with their dorsal surface facing up and sealed with a coverslip. Imaging was performed using an Axio Zoom.V16 microscope with a 2.3× 0.57 NA objective, a FS 38 HE filter set (with excitation at 470/40 nm and emission at 525/50 nm), and a Teledyne Prime 95B camera. Images were acquired continuously for either 6 or 10 min at a frequency of 20 Hz, with final magnifications of either 80× or 260× and a 2 μm depth of field to keep NMJs in focus during slight movements. High-power (260×) imaging data were collected from dorsal muscles 1, 2, 9, and 10 in either segment A1 or A2, whereas low-power (80×) imaging captured SynapGCaMP6f activity from segments A2, A3, and A4, where background autofluorescence from the gut and other organs minimally interfered with SynapGCaMP6f signal. The anterior segments provided clearer images with less autofluorescence and light scattering. VNC imaging was performed at the low-power magnification and encompassed the entire VNC. Only larvae showing sustained activity were included in the analysis, while those with excessive movement or focus issues were excluded.

### Quantification and Statistical Analysis.

#### Optical quantal image analysis.

Quantal image analysis was performed using techniques described in previous work (32, 42). In summary, optical quantal image analysis was conducted using custom MATLAB routines. The process involved filtering images to reduce noise, separating them into Ib and Is regions based on fluorescence, and correcting for motion and bleaching. Stimulus trials were excluded if they were out of focus, moved, or the stimulus failed.

For automatic detection of ΔF spots, templates were created from manually identified spots. ΔF response images were generated, and participating pixels were identified based on correlation with the template response. Release probability maps were constructed by identifying ΔF spot centers across multiple trials and color-coded to show the frequency of spot centers. Individual release sites were manually identified, and release probability values were calculated by dividing the number of ΔF spot centers by the number of trials. Quantal density, an unbiased metric, was calculated by averaging the number of ΔF spot centers over all trials and normalizing by the imaged area.

#### Behavioral analysis.

Movies were processed using custom MATLAB scripts to assess two phases of larval locomotion—an active crawling phase and a reorientation phase, as described previously (47). During the active crawling phase, a larva moves along a relatively straight and persistent trajectory. These periods of active crawling are interspersed with phases of reorientation, where the larva remains in the same spot bending and sweeping its head back and forth. As a result, these reorientation phases often lead to pronounced turning events.

Larval trajectories were determined using a 2D centroid tracking algorithm. To minimize noise in the crawling path, centroids within 1 mm of each other over a 2-s window were merged by computing the center of mass. Stride length was calculated by measuring the distance between each of these successive center-of-mass values. Reorientation events were identified by fitting a third-degree polynomial along the length of the larva’s body and analyzing its curvature using LineCurvature2D.m function in MATLAB (67). Curvature values were assigned to a larva for each frame to create a distribution across the entire movie. A reorientation event was counted when body curvature exceeded the 75% quartile of the distribution and lasted at least 500 ms. Turning angles were calculated by measuring the angle between two successive phases of active crawling separated by a single reorientation event. Velocity was calculated using the total distance crawled throughout the experiment divided by the recording duration. Time spent during reorientation events was calculated from the number of centroids used to calculate a smoothed point scored as a reorientation event. Time spent crawling on a continuous trajectory was calculated from the time in between reorientation events, excluding periods where the larvae stopped crawling.

#### In vivo image analysis.

In vivo image analysis was performed as previously reported using custom MATLAB routines (32). In summary, to stabilize the moving and contracting NMJs in vivo, the images were processed through a series of registration steps. Each recording typically included multiple Ib and Is NMJ pairs, which were processed individually. For high-power imaging (260× magnification) of the NMJ using either muscle-driven SynapGCaMP6f or motor-neuron-driven GCaMP6f, initial corrections for coarse, rigid translational movements were achieved by calculating x- and y-displacements to maximize the 2D cross-correlation between successive frames. Subsequent registration of the NMJs within the tracked areas involved affine and diffeomorphic nonlinear (Demon) transformations (MATLAB Image Processing Toolbox Release R2022b). A high-intensity frame, featuring both active Ib and Is NMJs, served as the reference for these corrections, which adjusted for local shape changes during muscle contractions. Fluorescence data were obtained from manually defined regions of interest around the NMJs or cell bodies in the VNC. For lower-power (80×) and VNC imaging, only the translation and affine registration steps were performed.

To isolate the synaptic fluorescence changes, spatially uniform muscle fluorescence signals (Fm) and any changes in background fluorescence were subtracted from the total fluorescence before calculating Ib and Is ΔF/F. This was achieved by calculating the mean fluorescence intensity from a region in proximity to the NMJs yet free of any synapses. While bleaching was minimal under these conditions, subtracting muscle fluorescence also accounted for any gradual bleaching that occurred during extended recordings. This approach ensured that the synaptic component of the SynapGCaMP6f or GCaMP6f fluorescence changes was precisely isolated in the complex in vivo environment of a behaving larva.

For either NMJ or VNC imaging, the onsets of evoked activity bouts were defined when either SynapCaMP6f or GCaMP ΔF/F surpassed 10% of the baseline level, whereas the ends of bouts fell below this threshold. Evoked activity bouts were manually verified by examining individual movies. Bout durations were calculated using the length in seconds of each activity bout, and the time to max was calculated from the start of the bout to maximum ΔF/F of the bout. The contraction displacement per segment was measured from the degree of translation from the center of mass of the NMJ being registered. Intersegmental bout delay is calculated from time of onset of one bout in a segment to the onset of the successive bout in the neighboring segment during P → A peristaltic waves. Overlap time between adjacent segments was measured when both NMJs from two neighboring segments were simultaneously active. Mean bout amplitude was calculated from measuring the average ΔF/F of the entire bouts. For high-power imaging (260×), the calculations for integrated fluorescence and muscle contraction magnitude were described in detail earlier (32). Fraction-time in evoked state is calculated by dividing the total time of evoked activity by the total time of the acquired movie.

## Supplementary Material

Appendix 01 (PDF)

Movie S1. Larval behavior tracking and curvature analysis, related to Figure 1.

Movie S2. Imaging at low magnification depicting forward-crawling peristaltic wave.

Movie S3. SynapGCaMP6f imaging of A4-A2 hemi-segments, related to Figure 3.

Movie S4. In vivo SynapGCaMP6f imaging at high magnification of Ib and Is NMJs, related to Figure 4.

Movie S5. SynapGCaMP6m imaging of PMSI neurons in VNC, related to Figure 5.

## Data Availability

Source data and analysis scripts included in the article have been deposited in the Zenodo repository (68). All other data are included in the manuscript and/or supporting information.
