# Supplementary material for: Behavioral resilience via dynamic circuit firing homeostasis
Source: Proc Natl Acad Sci U S A. 2025 Apr 29;122(18):e2421386122. doi: 10.1073/pnas.2421386122 (PMC12067288; doi:10.1073/pnas.2421386122)
Supplement: Supplementary file 1 — Appendix 01 (PDF) [file pnas.2421386122.sapp.pdf]

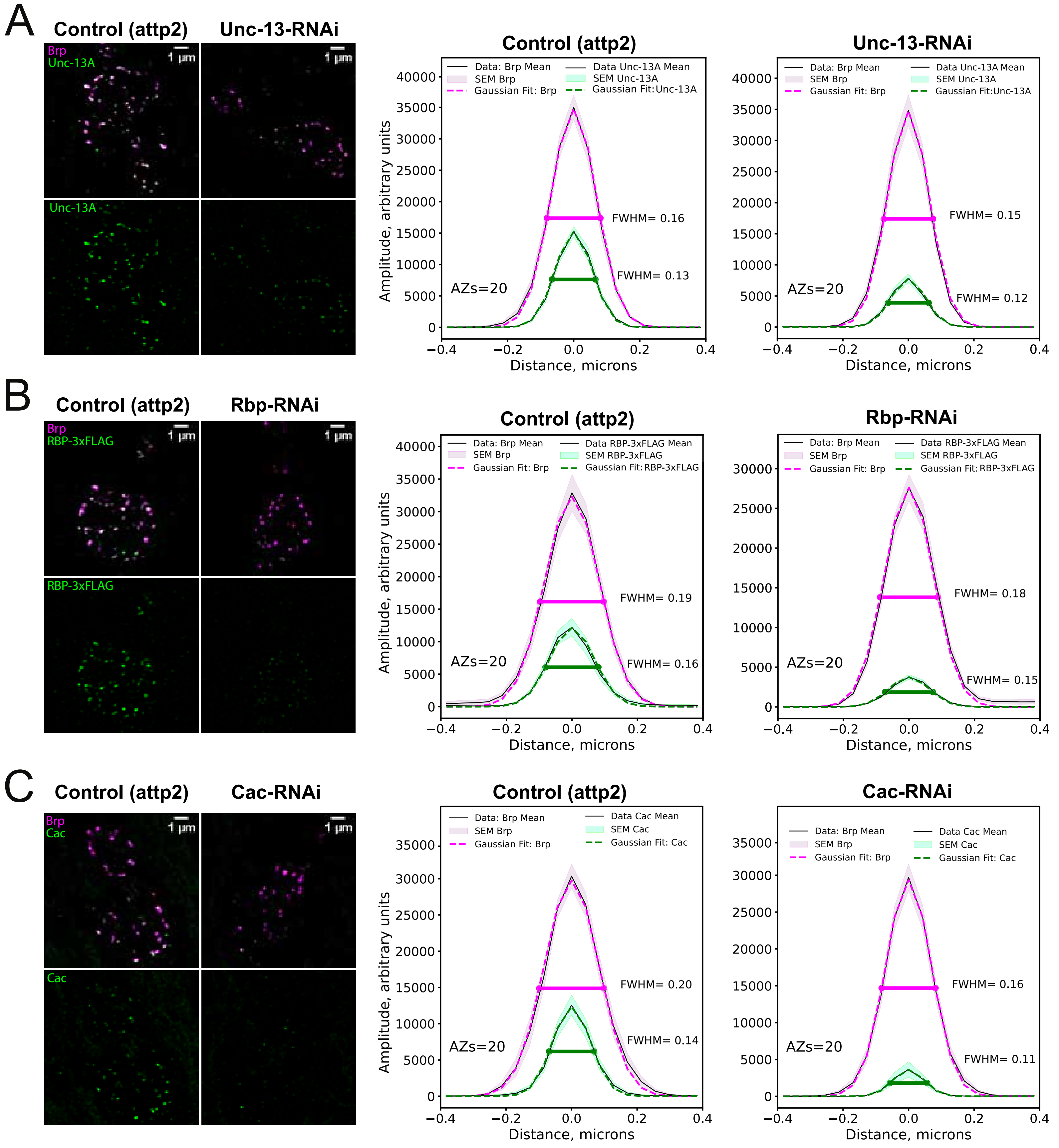

Figure S1. Antibody staining confirms presynaptic RNAis knock down expression of the targeted protein at active zones. (A) Representative terminal boutons for control (attp2(empty)) and Unc-13 RNAi. Imaging synaptic proteins was done using Airyscan and deconvolution, and the maximum intensity frame in the series is displayed (see methods). Synaptic full width half maxima and peak fluorescence intensity were calculated by Gaussian fitting the mean fluorescence intensity, for 20 synaptic active zones per terminal bouton, by bisecting individual active zones in their maximum intensity frame. Mean fluorescence values, SEM, and Gaussian fit of the means are displayed. (B) Representative terminal boutons for control (attp2) and Rbp-RNAi. Images were acquired and processed using identical settings as in (A). (C) Representative terminal boutons for control (attp2) and Cac-RNAi. Images were acquired and processed using identical settings as in (A).

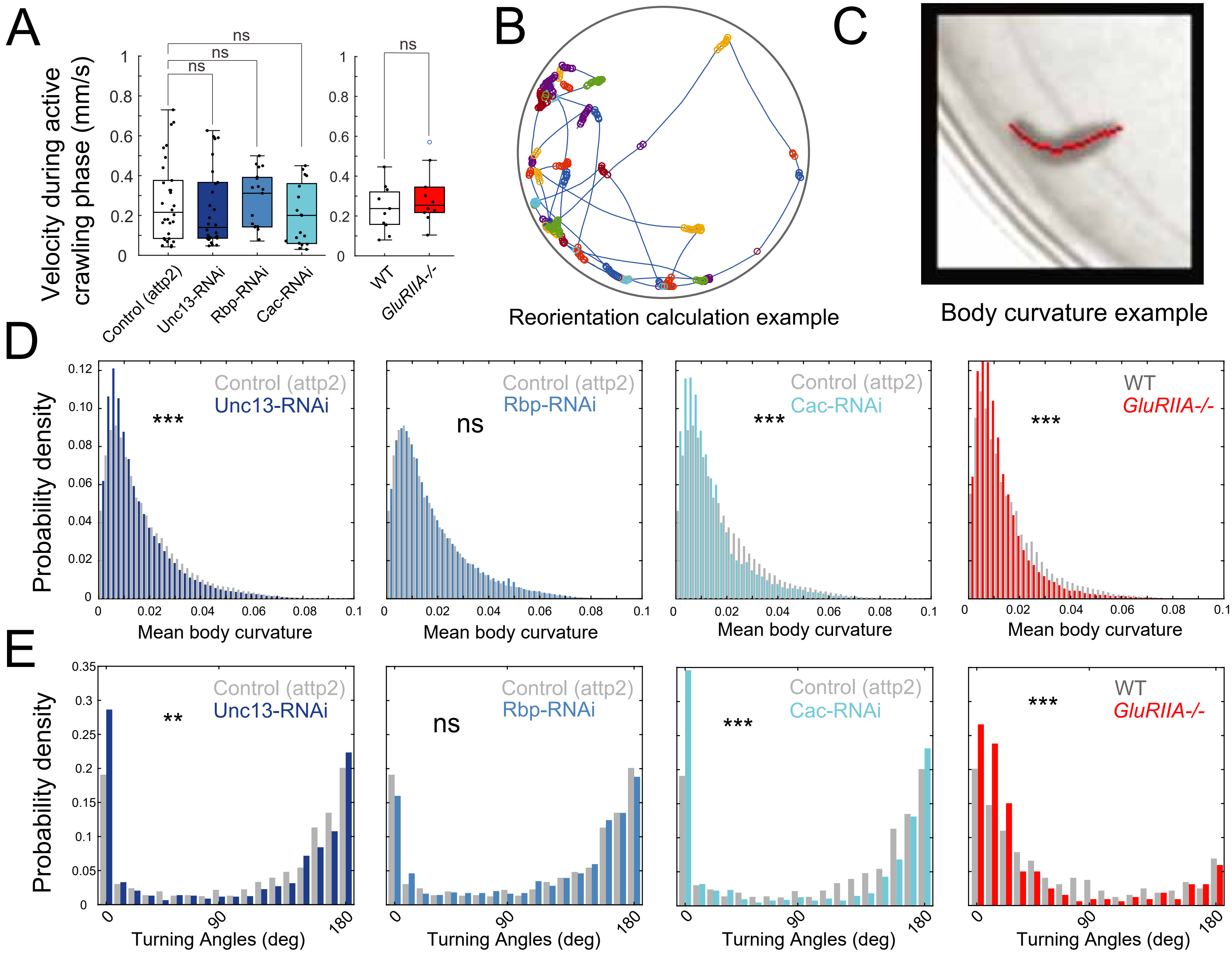

A
B
C
D
E
Velocity during active crawling phase (mm/s)
ns
ns
ns
ns
Control (attp2)
Unc13-RNAi
Rbp-RNAi
Cac-RNAi
WT
GluRIIA-/-
Reorientation calculation example
Body curvature example
Probability density
Mean body curvature
Control (attp2)
Unc13-RNAi
***
Rbp-RNAi
ns
Cac-RNAi
***
WT
GluRIIA-/-
***
Turning Angles (deg)
**
ns
***
***

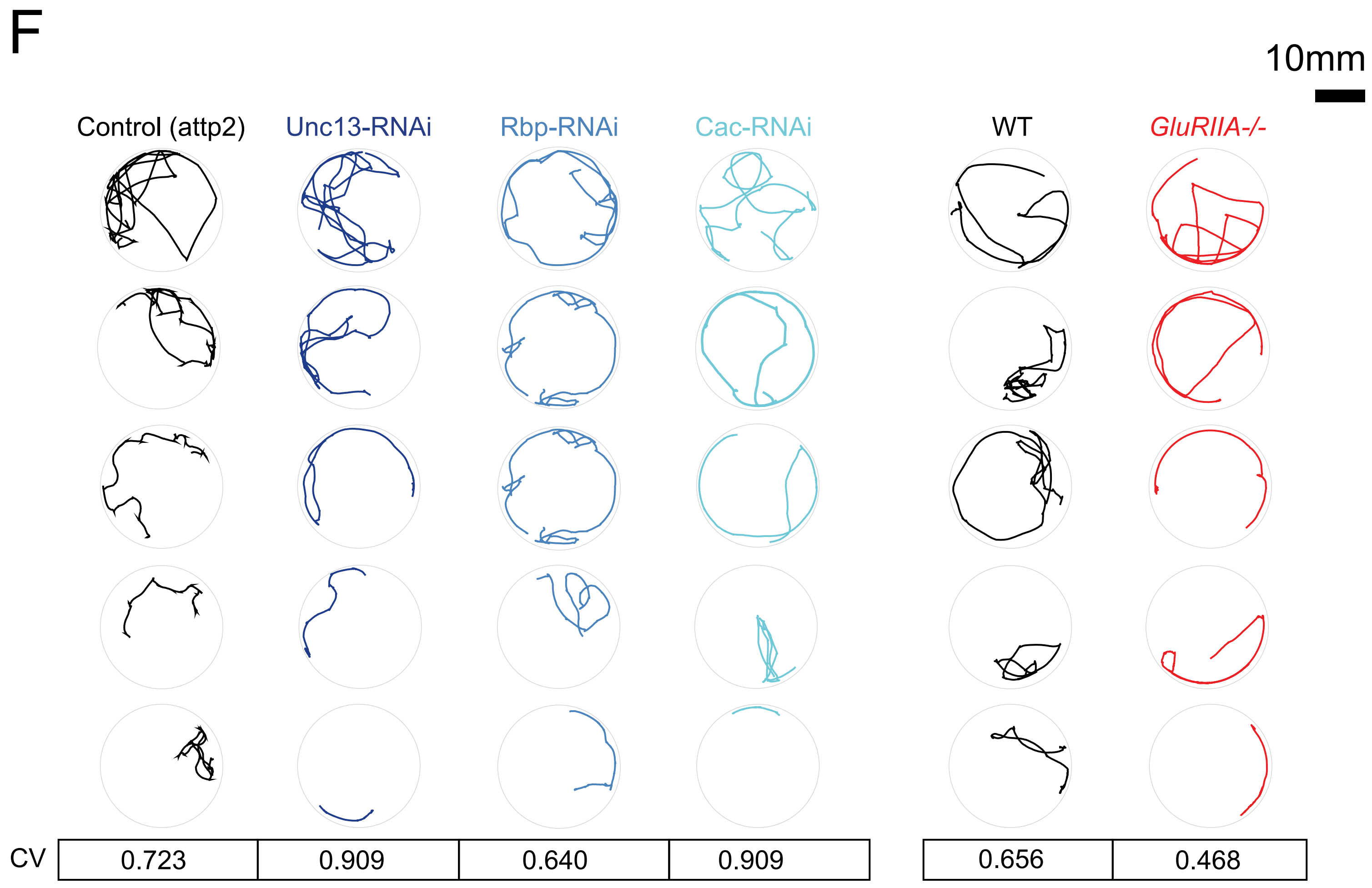

Figure S2. Effect on crawling agility of perturbation of presynaptic release machinery or postsynaptic glutamate receptor. (A) Velocity during the active crawling phases. (B) Example of active crawling phases (blue lines) punctuated by reorientation phases (circles). (C) Representative spline (red) fit along the body axis of the larva. (D) Probability densities of mean body curvatures for RNAis compared to attp2(empty) control and GluRIIA mutant compared to WT control. (E) Distributions of turning angles from 0 to 180 degrees. (F) Additional representative plots of crawling trajectories, with the coefficient of variation (CV) for velocities across all animals within each group reported below. Points are average value for each larva. Box plots depict median, the lower and upper quartiles, any outliers (open circles, computed using the interquartile range), and whiskers encompass the minimum and maximum values that are not outliers. Statistical comparisons Mann-Whitney test (A,D,E) *p<0.0.5; **p<0.01; ***p<0.001.

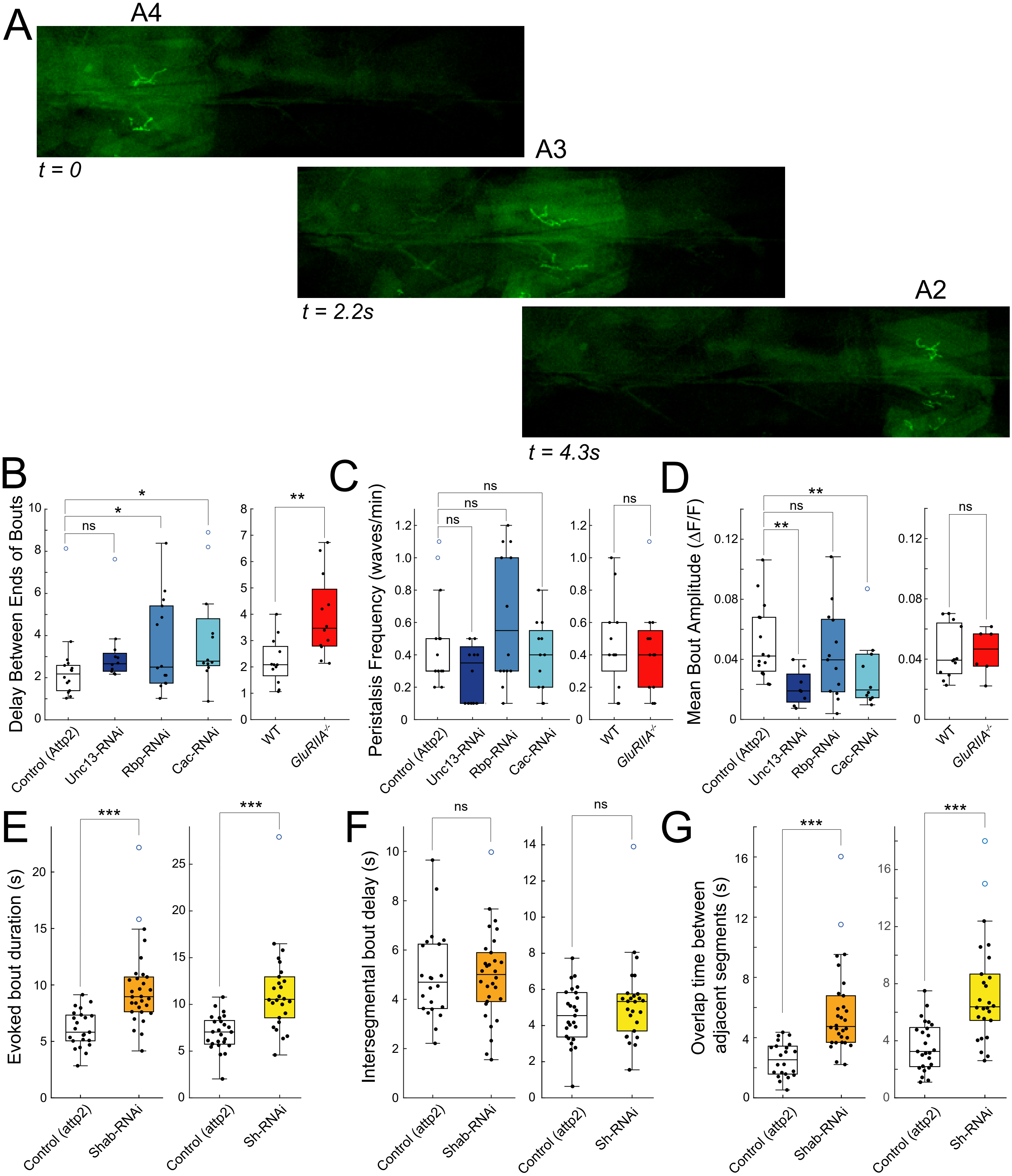

Figure S3. Extended properties of posterior-to-anterior (P → A) peristaltic bouts. (A) Representative P → A wave from control movie (20 fps). (B) Average delay between ends of bouts in neighboring segments during P → A peristaltic waves. (C) P → A peristaltic wave frequency. (D) P → A mean bout amplitude. (E-G) Evoked bout duration, intersegmental bout delay, and overlap time between adjacent segments for OK6>Shab-RNAi (orange) and OK6>Sh-RNAi (yellow) compared to attp2(empty) control (white). (B-G) Points are average value for each larva. Box plots depict median, the lower and upper quartiles, any outliers (open circles, computed using the interquartile range), and whiskers encompass the minimum and maximum values that are not outliers. Statistical comparisons Mann-Whitney test (B-G) *p<0.0.5; **p<0.01; ***p<0.001.

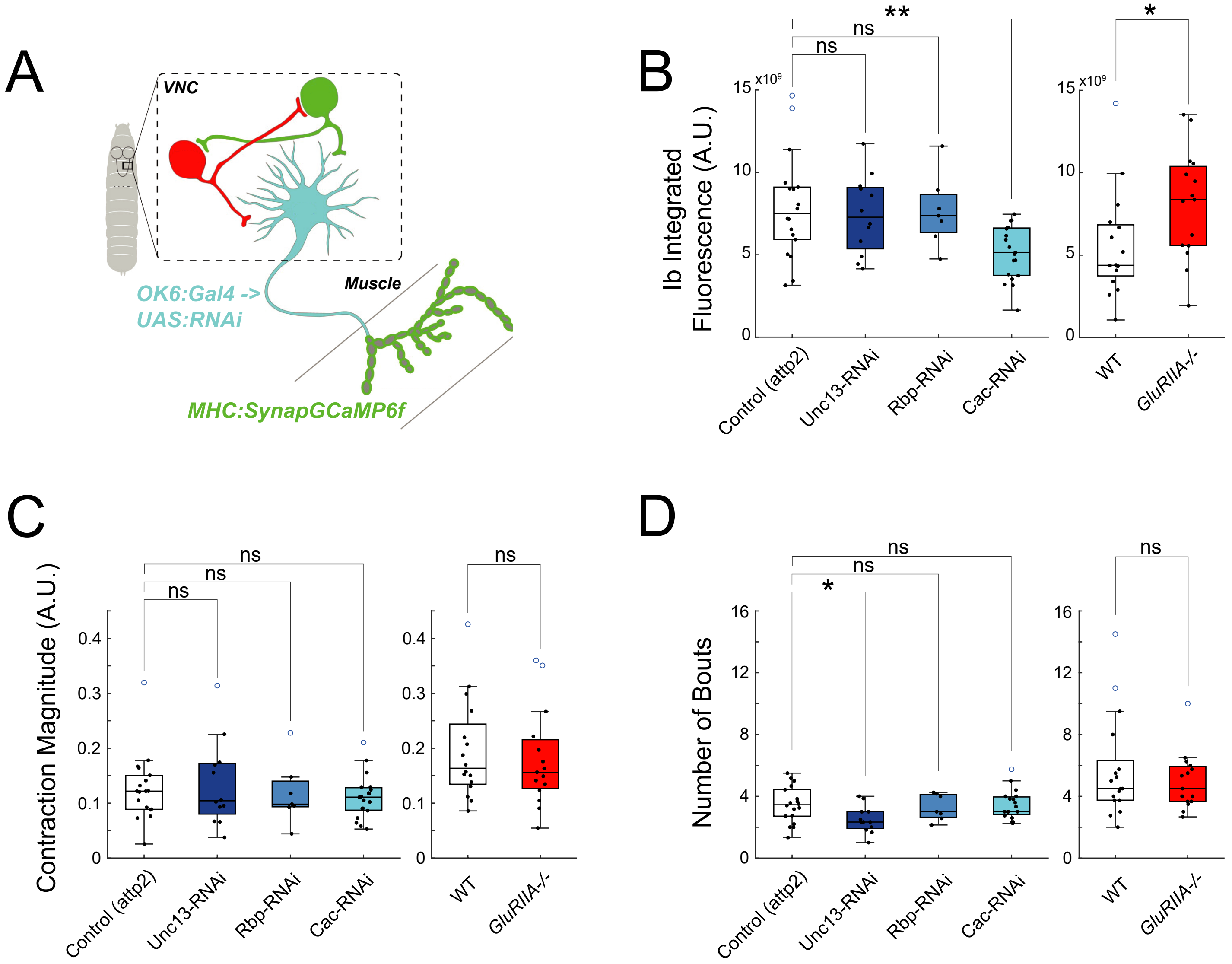

Figure S4. Extended properties of Ib and Is inputs in segment A1. (A) Schematic of SynapGCaMP6f imaging. (B) Total MN synaptic transmission measured with postsynaptic SynapGCaMP6f integrated fluorescence. (C) Contraction magnitude as represented by the determinant of the affine transmformation used in image registration. (D) Number of bouts over the recording period (6 minutes). Points are average value for each larva. Box plots depict median, the lower and upper quartiles, any outliers (open circles, computed using the interquartile range), and whiskers encompass the minimum and maximum values that are not outliers. Statistical comparisons Mann-Whitney test (B-D) *p<0.0.5; **p<0.01; ***p<0.001.

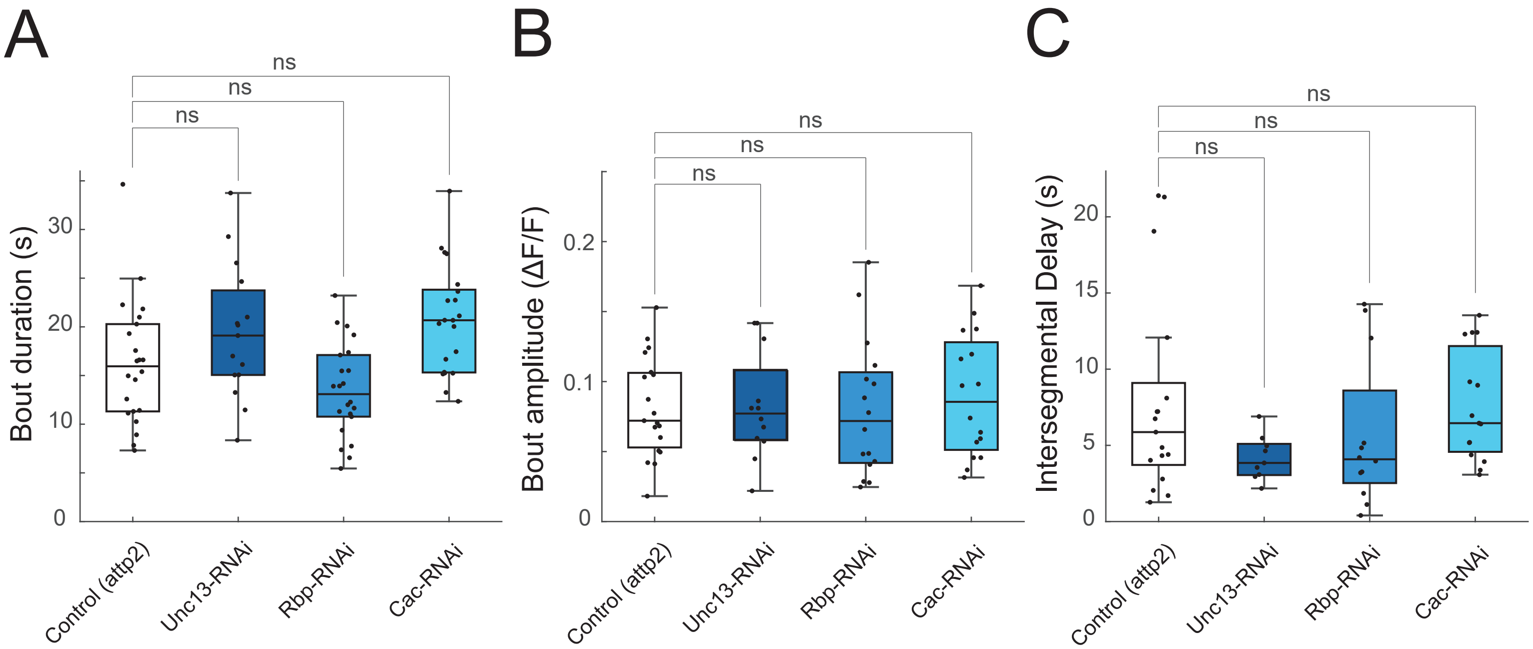

Figure S5. Extended results for effect of weakened MN to muscle synapse on activity of PMSI inhibitory pre-motor neurons. (A) Bout duration. (B) Bout amplitude. (C) Intersegmental delay between bout onset during P → A peristaltic waves. Box plots depict median, the lower and upper quartiles, any outliers (open circles, computed using the interquartile range), and whiskers encompass the minimum and maximum values that are not outliers. Statistical comparisons Mann-Whitney test *p<0.0.5; **p<0.01; ***p<0.001.

Supplemental Movie Legends

Movie S1. Larval behavior tracking and curvature analysis, related to Figure 1.

Movie S2. Imaging at low magnification depicting forward-crawling peristaltic wave.

Movie S3. SynapGCaMP6f imaging of A4-A2 hemi-segments, related to Figure 3.

Movie S4. In vivo SynapGCaMP6f imaging at high magnification of Ib and Is NMJs, related to Figure 4.

Movie S5. SynapGCaMP6m imaging of PMSI neurons in VNC, related to Figure 5.
